# Regional anaesthesia is associated with less patient satisfaction compared to general anaesthesia following distal upper extremity surgery: a prospective double centred observational study

**DOI:** 10.1186/s12871-019-0789-4

**Published:** 2019-07-02

**Authors:** Wouter Droog, Sanne E. Hoeks, G. Peter van Aggelen, D-Yin Lin, J. Henk Coert, Robert Jan Stolker, Eilish M. Galvin

**Affiliations:** 1 Department of Anaesthesia, Erasmus University Medical Centre Rotterdam, P.O. Box 2040, 3000 CA Rotterdam, The Netherlands; 2 Department of Anaesthesia, Franciscus Gasthuis & Vlietland, P.O. Box 10900, 3004 BA Rotterdam, The Netherlands; 3 Department of Plastic Surgery, University Medical Centre Utrecht, P.O. Box 85500, 3508 GA Utrecht, The Netherlands

**Keywords:** Patient satisfaction, Anaesthesia, conduction, Anaesthesia, general, Patient care

## Abstract

**Background:**

Patient satisfaction is a well-established indicator to evaluate the quality of medical care and there is an increasing support for the use of patient-reported experience measures (PREMs) to evaluate satisfaction. To anesthetize the upper limb for surgery, both general and regional plexus anaesthesia are appropriate techniques. However, the best technique in the anaesthesiologist’s perspective might not necessarily result in the highest patient satisfaction. The aim of this study is to investigate patient satisfaction following general and regional anaesthesia, and to identify areas where anaesthesiologists can focus on improving patient care.

**Methods:**

Patients scheduled for elective distal upper extremity surgery under either general or regional plexus anaesthesia were prospectively included. On the first postoperative day, patient satisfaction and main reason for dissatisfaction with the anaesthesia technique were investigated during a telephone interview.

**Results:**

Of the 243 patients included in the current study, 79.8% report being “fully satisfied” with their anaesthesia technique. 32.1% of the patients who received regional anaesthesia reported not feeling “fully satisfied”. This figure is 5.5% following general anaesthesia. Main reason for dissatisfaction following regional anaesthesia are reported as “insufficient anaesthesia prior to surgery”, and “the discomfort of having a long-lasting insensate extremity postoperatively”.

**Conclusions:**

Following regional plexus anaesthesia, a third of the patients are not “fully satisfied”. To optimize patient satisfaction following regional anaesthesia techniques, we advocate stronger focus on patient counselling preoperatively, addressing the issues of block failure and prolonged postoperative sensory and motor block.

## Background

Patient satisfaction is a well-established indicator to evaluate the quality of medical care and it is an important tool for prompting improvements in clinical care [1–3]. There is increasing support for the use of patient-reported experience measures (PREMs) and The American Society of Anesthesiologists (ASA) recognizes the importance of assessing patient satisfaction and experience [4]. In general, patient satisfaction with anaesthesia care is reported as very high [3, 5], but during times of rising patient expectations, areas for potential improvement are worthy of investigation.

For distal upper extremity surgery a variety of anaesthetic techniques are available to anesthetize the upper limb; general anaesthesia and regional (plexus) blocks are commonly applied techniques. Both techniques have their advantages and disadvantages, and the choice of technique largely depends on the planned surgery and patient’s overall health status and preferences. The anaesthesiologist is faced with the task of choosing the “best” anaesthesia technique for an individual patient. In clinical practice however, the best technique in anaesthesiologist’s perspective, might not necessarily result in the highest patient satisfaction. Therefore, the aim of the current study is to investigate patient satisfaction following both general and regional plexus anaesthesia. The secondary aim of this study is to identify areas for clinical practice improvement.

## Methods

This study was carried out by the Departments of Anaesthesia and Plastic Surgery of two hospitals; a large university hospital and a smaller teaching hospital. The study formed part of a prospective study on nerve injury following distal upper extremity surgery including a total of 335 patients. For this analysis on patient satisfaction following general and regional plexus anaesthesia, we only included those patients scheduled for elective upper extremity surgery under these two anaesthesia techniques. We excluded patients operated under other forms of anaesthesia techniques, such as Bier block or distal individual nerve blocks, and those with a planned combination of general and regional anaesthesia. All patients included gave written informed consent and were 18 years and older. The local Medical Ethics Committee of both hospitals reviewed and approved this study (October 11, 2012, number 2012–327).

### Preoperatively

All patients scheduled for elective upper extremity surgery were referred to the anaesthesia outpatient clinic by the treating plastic hand surgeon. Structured history-taking and clinical examination was used to evaluate overall health status. The choice between general or regional anaesthesia for the planned surgery was made on the basis of patient factors, patient preferences, and the planned procedure. Patients were verbally informed about the planned anaesthetic technique and possible complications. If patients were medically suitable candidates for both regional and general anaesthesia, they were informed on both techniques and in shared discussion the actual technique was chosen. All patients agreed on the proposed anaesthesia technique.

### Intraoperatively

Data on the anaesthetic technique and medication used were collected using the computerized Hospital Information System. The technical performance of the anaesthesia technique and medication used was at the discretion of the treating anaesthesiologist. The same applies for the use of ultrasound, nerve stimulator, needle type and diameter, and other equipment necessary for the regional block performance. Regional plexus blocks were performed using ropivacaine 7.5 mg/mL, mepivacaine 2 mg/mL, lidocaine 10 mg/mL, prilocaine 10 mg/mL or a combination of these agents. Dose (mg/kg) and volume (mL) of local anaesthetic used, was determined by the treating physician. Analgesia and sedation used during performance of regional anaesthesia or during surgery, consisted of one or a combination of alfentanil, sufentanil, propofol, and midazolam. Targeted sedation level during block performance was Ramsay sedation scale level 2, which means that patients are cooperative, orientated and tranquil [6].

All patients were scheduled for day-care surgery and all patients were expected to be discharged on the day of surgery. Before discharge home, patients had to meet local post-operative discharge criteria for day-care surgery. These criteria include: stable vital signs, proper orientation, able to drink/eat/void/dress/walk (without assistance), minimal nausea/vomiting/pain, and the presence of an adult escort home. In all patients, postoperative medication included acetaminophen plus a nonsteroidal anti-inflammatory drugs and/or opioids (tramadol or oxycodone).

### Postoperatively

On the first postoperative day, all patients received a telephone call from a nurse from the hospital ward, assessing postoperative pain scores, patient satisfaction, and main reason for dissatisfaction. The day-one postoperative questionnaire used in this study was developed by the research team (Appendix). Before analysis of the data, main reasons for dissatisfaction were clustered. The nurse was not affiliated with the anaesthesia department and was not aware of the anaesthesia technique.

### Outcome

Satisfaction was measured on a three-point Likert rating scale, by asking patients to rate if they were “fully satisfied”, “partly satisfied”, or “dissatisfied” with their anaesthesia technique. A three-point scale was used to simplify identification of patients who were dissatisfied and those who were not. Comparable reasons for patient dissatisfaction were clustered into groups. Postoperative pain was measured using the Numeric Rating Scale (NRS). The NRS is a verbally administered 11-point numeric rating scale, on which a patient can report pain intensity ranging from 0 (“no pain”) to 10 (“worst pain imaginable”) [7]. All scores of four or higher were considered abnormal. All scores under four were labelled as “pain level acceptable to the patient”.

### Statistical analysis

Statistical analysis was performed using SPSS 25.0 (IBM Corporation, Armonk, NY, USA). Continuous variables were tested for normality of the distribution and were presented as mean (± SD) or median (+ IQR). Kruskal-Wallis and Mann-Whitney U tests were used for continuous variables. For categorical variables the Fisher’s exact test was used. A *P*-value of 0.05 or less was considered to be statistically significant.

## Results

A total of 335 patients scheduled for elective upper extremity surgery were consecutively screened and asked to participate in the study. After excluding the patients that did not meet the inclusion criteria for the current analysis (*n* = 77), and patients who were not contactable day 1 post surgery (*n* = 15), a total of 243 patents were investigated. Of these 243 patients 134 received regional and 109 general anaesthesia prior to surgery.

Patient and surgical characteristics, pain-scores (NRS), and patient satisfaction scores are summarized in Table 1. Details on anaesthesia technique and patient satisfaction are shown in Table 2.

**Table 1:** Patient and Surgical Characteristics and Satisfaction Scores

	Total	Fully Satisfied	Partly Satisfied	Dissatisfied	*P*-value
Total	243	194 (79.8%)	36 (14.8%)	13 (5.3%)	
Gender					0.425
Female (*n*)	131	101	23	7	
Male (*n*)	112	93	13	6	
Age (yrs.)^a^					0.482
median	52.0	52.5	49.5	47.9	
(IQR)	(36.6–61.8)	(37.8–62.9)	(32.5–58.0)	(41.9–57.8)	
BMI (kg/m^2^)^a^					0.693
median	24.9	24.9	24.9	22.9	
(IQR)	(23.0–27.4)	(23.4–27.5)	(22.9–26.9)	(21.5–29.8)	
ASA-classification^b^					0.902
ASA 1 (*n*)	116	92	17	7	
ASA 2 (*n*)	118	95	18	5	
ASA 3 (*n*)	9	7	1	1	
NRS-Score^a^					
median	3	3	5	7	0.011
(IQR)	(1–6)	(1–6)	(1.25–7)	(2–8.5)	
NRS > 4	118	88	22	8	0.139
NRS < 4	125	106	14	5	
Type of Surgery^c^					
Arthrodesis/arthroplasty (*n*)	37	27	4	6	
Carpal Tunnel Syndrome (*n*)	8	8	0	0	
Cubital Tunnel Syndrome (*n*)	2	1	0	1	
Dupuytren’s contracture (*n*)	57	48	7	2	
Finger-joint replacement (*n*)	1	1	0	0	
Ganglion cyst removal (*n*)	5	3	2	0	
Ligament repair surgery (*n*)	8	7	1	0	
Neuroma excision (*n*)	3	3	0	0	
Placement of osteosynthesis material (*n*)	4	4	0	0	
Proximal row carpectomy (*n*)	3	1	2	0	
Quervain’s release surgery (*n*)	3	2	1	0	
Removal of osteosynthesis material (*n*)	7	5	2	0	
Tendon repair surgery (*n*)	13	11	2	0	
Tenolysis (*n*)	5	4	1	0	
Trigger finger release (n)	2	2	0	0	
Ulnar nerve transposition (*n*)	9	8	1	0	
Wrist arthroscopy (*n*)	21	12	8	1	
Miscellaneous (*n*)	55	47	5	3	
Tourniquet use^c^					
Yes (*n*)	235	186	36	13	
No (*n*)	8	8	0	0	
Duration of Tourniquet use (minutes)					
median	46.5	45	50	46	0.875
(IQR)	(30–65)	(30–65)	(31–61)	(37.5–64.5)	

^*a*^ *Age, BMI and NRS-Score are not normally distributed and therefore presented as ‘median (IQR)’*

^*b*^ *ASA-classification (class 1–6), according to The American Society of Anesthesiologists (ASA) physical status classification system*

^*c*^ *Due to the small numbers in some cells, no statistical analyses was performed on type of surgery details*

**Table 2:** Anaesthesia Characteristics and Satisfaction Scores

	Total	Fully Satisfied	Partly Satisfied	Dissatisfied	*P*-value
Total	243	194 (79.8%)	36 (14.8%)	13 (5.3%)	
Type of Anaesthesia					< 0.001
General Anaesthesia	109	103	5	1	
Regional Anaesthesia^a^	134	91	31	12	
Adjuvants used during regional anaesthesia procedure^b^	134	91	31	12	
Nerve Stimulator	12	7	4	1	
Ultrasound	29	20	5	4	
Nerve Stimulator & Ultrasound	93	64	22	7	
Local anaesthetic used during regional anaesthesia procedure^b^	134	91	31	12	
Ropivacaine (7.5 mg/mL)	116	78	29	9	
Mepivacaine (2 mg/mL)	7	5	1	1	
Mix of Ropivacaine and Mepivacaine	6	6	0	0	
Other^c^	5	2	1	2	
Medication during regional anaesthesia procedure	134	91	31	12	0.392
Yes	81	52	22	7	
No	53	39	9	5	
Medication administered during surgical procedure ^d^	117	84	26	7	0.018
Yes	33	19	13	1	
No	84	65	13	6	

^*a*^ *Regional Anaesthesia Details (n = 134): Pippa Block 1, Interscalene Brachial Plexus Block 3, Supraclavicular Brachial Plexus Block 18, Axillary Block 112*

^*b*^ *Due to the small numbers in some cells, no statistical analyses was performed on these regional anaesthesia details*

^**c**^*Other local anaesthetics used were: Prilocaine (10 mg/mL), Lidocaine (10 mg/ml*) *or a mix of Lidocaine and Ropivacaine*

^d^ *Medication during procedure was only administered in the 117 patients receiving regional anaesthesia alone, without the 17 patients who received general anaesthesia due to insufficient regional anaesthesia*

One hundred ninety-four patients of a total of 243 patients (79.8%) reported being “fully satisfied” with their anaesthesia technique (Table 1). 36 patients are “partly satisfied” (14.8%), and 13 patients (5.3%) are “dissatisfied” with their anaesthesia technique (Table 1). There is no difference in satisfaction scores comparing for gender, age, BMI, or ASA-classification (Table 1). Median pain scores are statistically significantly higher in patients who are “dissatisfied” in comparison to patients who are “partly satisfied” or “fully satisfied” in both groups (Table 1). Median preoperative pain scores are 3 (0 – 6) and 4 (0 – 7) in the general and regional anaesthesia group respectively (Table 3). Median postoperative pain scores are 3 (1–5) and 4 (1–7) following general or regional anaesthesia respectively (Table 3). There is no statistically significant difference in median pre- and postoperative pain scores between the general and regional anaesthesia group (Table 3).

**Table 3:** NRS-Score and Anaesthesia Technique

	Total	General Anaesthesia	Regional Anaesthesia	*P*-value
Number	243	109	134	
Preoperative NRS-Score				
median	4	3	4	0.201
(IQR)	(0–7)	(0–6)	(0–7)	
NRS > 4	124	50	74	0.147
NRS < 4	119	59	60	
Postoperative NRS-Score				
median	3	3	4	0.139
(IQR)	(1–6)	(1–5)	(1–7)	
NRS > 4	118	50	68	0.450
NRS < 4	125	59	66	

*Data is presented as number and ‘valid percentage’*

Patients following regional anaesthesia have lower levels of being “fully satisfied” with their anaesthesia technique than those following general anaesthesia (Table 2). Of the total of 134 patients who received regional anaesthesia as the primary anaesthesia technique, 43 patients (32.1%) are not “fully satisfied” versus 6 patients of a total of 109 (5.5%) general anaesthesia patients (Table 2). Due to insufficient anaesthesia prior to surgery, conversion from regional to general anaesthesia occurred in 17 of the 134 patients (13%) and additional or rescue blocks were performed in 12 of the 134 patients (9%).

Main reasons for dissatisfaction with regional anaesthesia in this study are: insufficient anaesthesia prior to surgery, and patient’s discomfort with their insensate and uncontrollable extremity postoperatively (Table 4). 14 patients do not reveal details on their reason for dissatisfaction (Table 4). In the 134 patients who received regional plexus anaesthesia, medication (sedatives/analgesics) administered during block placement procedure did not result in more patients being “fully satisfied” (Table 2).

**Table 4:** Patient Comments for not being “fully satisfied” with their Anaesthesia Technique

Comment	Number
Insufficient regional anaesthesia (resulting in additional blocks, or conversion to general anaesthesia)	18
Patient discomfort with the insensate and uncontrollable extremity postoperatively	6
Symptoms of local anaesthetic toxicity	1
Pain during regional anaesthesia procedure	2
Pain following regional anaesthesia wear-off	3
Uncomfortable sensations during wear-off of regional anaesthesia (e.g. tingling)	2
Horner’s syndrome	1
Nausea following general anaesthesia	2
No specific reason given	14
Total	49

## Discussion

In the current study, 79.8% of the 243 participants report being “fully satisfied” with the anaesthetic technique used during their surgery. Previous research shows that satisfaction with all aspects of health care is high (> 85%), and satisfaction with the anaesthetic care provided is often even higher (up to 96.8%) [3, 5]. Compared to these findings, patients in the current study are less frequently “fully satisfied”. This can partly be explained by the fact that the current study focuses specifically on anaesthesia technique used, as supposed to others who have focussed on quality of recovery after anaesthesia [3, 5]. Patient characteristics commonly associated with high satisfaction scores are: older age, male gender, and co-existing medical conditions (or ASA-class > 3) [3, 5, 8, 9]. These patient characteristics are of no influence on satisfaction scores in the current study.

Patients following regional anaesthesia have significantly lower levels of being “fully satisfied” in comparison to those following general anaesthesia. Following regional anaesthesia, 32.1% of the patients are not “fully satisfied” versus only 5.5% following general anaesthesia. Further evaluation of those patients who are not “fully satisfied” with their anaesthesia technique reveals two frequently reported complaints. Firstly, a feeling of insufficient anaesthesia prior to surgery. Obviously this is a reason for dissatisfaction; patients assume a fully working regional block, as this was communicated during the outpatient visit. Unfortunately, in 28 patients receiving regional anaesthesia, additional (rescue) blocks or conversion to general anaesthesia was needed.

The second most common reason given for lower satisfaction is patient’s discomfort with their insensate and uncontrollable extremity postoperatively. Prolonged postoperative analgesia is a frequently referenced as a major advantage of regional anaesthesia over general anaesthesia [10]. However, in the current study, the prolonged postoperative anaesthesia is a reason for not being “fully satisfied”. This finding highlights a mismatch between doctor’s opinion of a successful outcome and that of the patient. PREMs are therefore of utmost importance to evaluate outcome and improve quality of care.

In the current study, median pain scores are significantly higher in patients who are “dissatisfied” in comparison to patients who are or “partly satisfied” or “fully satisfied” following both general and regional anaesthesia. A correlation between higher pain scores and dissatisfaction would seem intuitive, but our findings indicate that this relationship is more complex. Of the 118 patients with high intensity pain scores (NRS > 4) a total of 88 patients (74.6%) are still “fully satisfied” with their anaesthesia technique. Thus, for some patients a certain level of postoperative pain is self-evident following surgery and lowering pain scores will not necessarily result in higher satisfaction scores.

How can we improve patient satisfaction in our daily clinical practice? In this particular study group, taking the limitations into account, regional anaesthesia results in less patients being fully satisfied compared to general anaesthesia. This finding suggests there is room for clinical practice improvement. In patients receiving regional anaesthesia, main reasons for dissatisfaction are: a feeling of insufficient anaesthesia prior to surgery, and patient’s discomfort with their insensate and uncontrollable extremity postoperatively. We therefore advocate that both these items are addressed in detail during pre- and postoperative counselling in those patients receiving regional anaesthesia. Of course, patients should be made aware of the risk of an insufficient regional block prior to surgery and the need for additional blocks, or sometimes conversion to general anaesthesia. Unfortunately, failure of regional anaesthesia techniques cannot always be prevented. Secondly, all regional anaesthesia patients should be counselled on prolonged postoperative sensory and motor block; because for some patients, this results in discomfort and a feeling of uncertainty. Duration of the motor/sensory block is highly variable and is difficult to predict with accuracy in individual patients [11]. We therefore strongly suggest that all patients are made aware of such variability in duration prior to block placement and before discharge home. Additionally, pain management should not end at discharge home, but an individual and intensive multi-modal analgesic plan should be in place for all patients.

A limitation of the current study, and many other studies on patient satisfaction, is the use of a simplistic three-point Likert rating scale to assess the highly complex and multidimensional concept of patient satisfaction [12]. Patient satisfaction is determined by many different variables, such as the quality of provided medical care, perceived outcomes, and preoperative expectations [2]. Proper assessment of satisfaction is therefore a complex task, and high satisfaction scores not necessarily reflect high quality of medical care [1, 3, 13]. To simplify this complex assessment, we entirely centred our attention on a single part of the total anaesthetic care given to a patient, and primarily focus on anaesthesia technique provided. Also, satisfaction scores in this current study are primarily used as an instrument to identify potentially modifiable factors associated with dissatisfaction, to provide an opportunity for clinical practice improvement.

Another limitation of the current study is the lack of randomization of patients between regional and general anaesthesia. This may introduces several biases, such as anaesthesiologists’ preference, patients’ preference, and bias through inclusion of patients who were medically not eligible for a specific anaesthesia technique. For example, if an anaesthesiologist prefers general anaesthesia, his exposure (and subsequently his success-rate) might be lower than in those who prefer regional anaesthesia. Also, satisfaction scores can be influenced in patient who prefer a specific anaesthesia technique, but find out this technique is (medically) not advisable.

A final limitation of the current study is the small sample size, with a wide variability in the types of surgical procedures, types of regional anaesthesia techniques, and low overall ASA-score. We chose to study a pragmatic cohort of patients undergoing elective distal upper extremity surgery, and therefore the present conclusions should be interpreted accordingly.

## Conclusions

For distal upper extremity surgery a variety of anaesthetic techniques are available to anesthetize the upper limb. The results of the current study suggest that one third of the patients are not “fully satisfied” following regional (plexus) anaesthesia techniques. To optimize patient satisfaction following regional anaesthesia techniques, we advocate focusing on patient counselling and more expansively addressing the issues of block failure and prolonged postoperative sensory and motor block.

## Data Availability

The datasets used and/or analysed during the current study are available from the corresponding author on reasonable request.

## Appendix

**Table 5:** Day-one postoperative Questionnaire (Translated from Dutch)

Question	Answer
Pain at the current moment (NRS)	0 / 1 / ... / 10 ^*^
Are you satisfied with the Anaesthesia Technique performed?	“fully satisfied” / “partly satisfied” / “dissatisfied”
What is your main reason for dissatisfaction?	… (open question)
Additional Comments	… (open question)

** NRS 0–10: 0 stands for “no pain” and 10 for “worst pain imaginable”*

## Abbreviations

ASA: American Society of Anesthesiologists
NRS: numeric rating scale
PREMs: patient-reported experience measures

## References

[1] Wu CL, Naqibuddin M, Fleisher LA (2001). Measurement of patient satisfaction as an outcome of regional anesthesia and analgesia: a systematic review. Reg Anesth Pain Med.

[2] Teunkens A, Vanhaecht K, Vermeulen K, Fieuws S, Van de Velde M, Rex S, Bruyneel L (2017). Measuring satisfaction and anesthesia related outcomes in a surgical day care center: a three-year single-center observational study. J Clin Anesth.

[3] Royse CF, Chung F, Newman S, Stygall J, Wilkinson DJ (2013). Predictors of patient satisfaction with anesthesia and surgery care: a cohort study using the postoperative quality of recovery scale. Eur J Anaesthesiol.

[4] Kingsley C, Patel S (2017). Patient-reported outcome measures and patient-reported experience measures. BJA Education.

[5] Myles PS, Williams DL, Hendrata M, Anderson H, Weeks AM (2000). Patient satisfaction after anesthesia and surgery: results of a prospective survey of 10,811 patients. Br J Anaesth.

[6] Ramsay MA, Savege TM, Simpson BR, Goodwin R (1974). Controlled sedation with alphaxalone-alphadolone. BMJ..

[7] Hawker GA, Mian S, Kendzerska T, French M (2011). Measures of adult pain: visual analog scale for pain (VAS pain), numeric rating scale for pain (NRS pain), McGill pain questionnaire (MPQ), short-form McGill pain questionnaire (SF-MPQ), chronic pain grade scale (CPGS), short Form-36 bodily pain scale (SF-36 BPS), and measure of intermittent and constant osteoarthritis pain (ICOAP). Arthritis Care Res (Hoboken).

[8] Coyle TT, Helfrick JF, Gonzalez ML, Andresen RV, Perrott DH (2005). Office-based ambulatory anesthesia: factors that influence patient satisfaction or dissatisfaction with deep sedation/general anesthesia. J Oral Maxillofac Surg.

[9] Cooper K, Kelley H, Carrithers J (1995). Perceptions of side effects following axillary block used for outpatient surgery. Reg Anesth.

[10] Klein SM, Buckenmaier CC (2002). Ambulatory surgery with long acting regional anesthesia. Minerva Anestesiol.

[11] Droog W, Lin DY, Huisman JS, Franssen FA, van Aggelen GP, Coert JH, Galvin EM (2017). Individual duration of axillary brachial plexus block is unpredictable: a prospective double centered observational study. Minerva Anestesiol.

[12] Barnett SF, Alagar RK, Grocott MP, Giannaris S, Dick JR, Moonesinghe SR (2013). Patient-satisfaction measures in anesthesia: qualitative systematic review. Anesthesiology..

[13] Capuzzo M, Gilli G, Paparella L, Gritti G, Gambi D, Bianconi M, Giunta F, Buccoliero C, Alvisi R (2007). Factors predictive of patient satisfaction with anesthesia. Anesth Analg.

